# An Improved Strapdown Inertial Navigation System Initial Alignment Algorithm for Unmanned Vehicles

**DOI:** 10.3390/s18103297

**Published:** 2018-09-30

**Authors:** Ya Zhang, Fei Yu, Wei Gao, Yanyan Wang

**Affiliations:** School of Electrical Engineering and Automation, Harbin Institute of Technology, Harbin 150001, China; yazhang@hit.edu.cn (Y.Z.); gaow@hit.edu.cn (W.G.); 16S101134@stu.hit.edu.cn (Y.W.)

**Keywords:** strapdown inertial navigation system, initial alignment, denoising, robust filter, Cubarure Kalman filter

## Abstract

Along with the development of computer technology and informatization, the unmanned vehicle has become an important equipment in military, civil and some other fields. The navigation system is the basis and core of realizing the autonomous control and completing the task for unmanned vehicles, and the Strapdown Inertial Navigation System (SINS) is the preferred due to its autonomy and independence. The initial alignment technique is the premise and the foundation of the SINS, whose performance is susceptible to system nonlinearity and uncertainty. To improving system performance for SINS, an improved initial alignment algorithm is proposed in this manuscript. In the procedure of this presented initial alignment algorithm, the original signal of inertial sensors is denoised by utilizing the improved signal denoising method based on the Empirical Mode Decomposition (EMD) and the Extreme Learning Machine (ELM) firstly to suppress the high-frequency noise on coarse alignment. Afterwards, the accuracy and reliability of initial alignment is further enhanced by utilizing an improved Robust Huber Cubarure Kalman Filer (RHCKF) method to minimize the influence of system nonlinearity and uncertainty on the fine alignment. In addition, real tests are used to verify the availability and superiority of this proposed initial alignment algorithm.

## 1. Introduction

The unmanned vehicle has been used as a platform for aerial photogrammetry, marine monitoring, geodetic surveying, hazard state investigation and security protection based on different sensors equipped on it. The navigation information of the unmanned vehicle provided by its navigation system, including the position, the velocity, the heading and the horizontal attitude, is the premise and guarantee of the normal working. The precision of navigation system is directly related to its track accuracy and safety.

The Global Navigation Satellite System (GNSS) can provide three-dimensional navigation information including position and velocity of everywhere on the earth with high-accuracy by utilizing satellite signals. Caused by signal blocking and multipath effect, GNSS has poor location accuracy in urban areas and forests normally [1]. By utilizing the acceleration and angular rate measured by gyroscopes and accelerometers, the Strapdown Inertial Navigation System (SINS) can calculate the vehicle’s velocity, position and attitude simultaneously [2]. Thus, compared with GNSS, SINS is an autonomous navigation system, which does not depend on external information, such as radio signal or radiates electromagnetic waves. Since SINS can track and reflect the vehicle’s maneuvering in time, the generated navigation data have the characteristics of high accuracy in a short-term, good stability and high data update rate. However, SINS is a time integration system and the navigation error is accumulated with time rapidly because of inertial sensors’ errors. Since the satellite navigation technology and the inertial navigation technology have their own advantages and limitations, therefore, in the navigation system of unmanned vehicles, GNSS and SINS are often integrated together to enhance the redundancy and accuracy [1,3,4,5].

With the continuous development of the autonomous navigation technology, higher requirements have been set about accuracy, size and reliability for navigation systems, whereas, to improve the accuracy of SINS, enhancing the manufacturing accuracy of inertial instruments simply will lead to a sharp increase in cost, so researching on the system algorithm has become the focus [6].

Since the initial parameters are determined by the initial alignment, the initial alignment is the prerequisite to ensure normal operation of the SINS for the unmanned vehicle [7,8]. In a general way, the initial alignment is divided into two stages which are the coarse alignment and the fine alignment  [9,10]. In the coarse alignment, the initial attitude matrix containing large errors is obtained roughly based on the output of the inertial sensors. In the fine alignment stage, an accurate attitude matrix has been calculated after the compensation of the attitude error estimated by the optimal estimation method [11].

Since the initial attitude is generally determined roughly by using the output of the inertial sensor in the coarse alignment process, the accuracy and reliability of the coarse alignment process largely depend on the accuracy of inertial sensors [9,12]. Limited by the unmanned vehicle’s payload, high-precision inertial sensors cannot be installed on it since there are strict requirements on the size and weight of its SINS. Thus, the output signal of inertial sensors contains a large amount of random noise due to limitations in the manufacturing process and package level [13]. Thus, it is necessary to denoise the output signal in order to suppress the impact of the random noise on coarse alignment.

The Wavelet Transform (WT) is a commonly used denoising method [14]. In the WT-based denoising method, the signal is decomposed into several coefficients by WT firstly, and then, according to containing noises or containing effective signals, coefficients are separated into two parts by the threshold. After removing coefficients that contain noises, the rest of the coefficients containing effective signals are reconstructed by utilizing the inverse WT method. Due to its advantage in multiresolution, the WT-based denoising method plays an important role in signal denoising. However, its performance entirely depends on the selection of wavelet basis functions, resulting in limitations in practical applications [15]. The Empirical Mode Decomposition (EMD) method decomposes the signal based on its time scale adaptively several times, and does not need the basis function for decomposition [16]. Moreover, the EMD method has great advantages in dealing with nonlinear and non-stationary random signals. In the EMD method, the generation of the envelope is the key technique and its quality will affect the decomposition result directly [17]. Due to its smoothness, the cubic spline method is one of the most commonly used envelope fitting methods. However, because of its non-monotony between two adjacent interpolations, overshoot or undershoot will be existed sometimes in practical applications. In severe cases, the intersection of the upper and lower envelopes or over-ranging the envelope will cause the failure of decomposition. To solve the above problems, B-spline (BS) interpolation is often used in place of the cubic spline interpolation. This decomposition method, named the BS-EMD method, can not only improve the computational efficiency but also preserve the local characteristics of the signal [18]. However, due to the uncertainty of the B-spline function, the endpoint effect will occur when dealing with finite time series, which seriously affects the reliability of the algorithm.

Methods to suppress the endpoint effect mainly include the suppression method based on the Mirror Extension (ME), the suppression method based on Support Vector Machine (SVM), the suppression method based on Neural Network (NN) integration and so on [19]. The ME-based suppression method extends the data only depending on the characteristics of the extreme points at the both ends, so the overall characteristics of the actual signal cannot be taken into consideration, inevitably affecting the accuracy of decomposition; The SVM-based suppression method completely depends on the selection of inner production function and the adjustment of parameters, and inaccuracy parameter selection also can cause inaccurate decomposition. The NN integration-based suppression method requires that individual subnetworks used for integration have large differences and the whole process takes a longer time compared with other algorithms. As a result, it is urgent to propose an effective signal denoising method to preprocess the inertial sensor data, enhancing the performance of coarse alignment.

In the process of fine alignment, the inertial sensors’ error is estimated and compensated using the optimal estimation algorithm to enhance the precision of initial attitude matrix [20]. The most commonly used estimations are based on a Kalman filter (KF) that can only handle linear systems and require that the noise statistics should be accurately known [21,22]. However, in the unmanned vehicle’s SINS, the performance of inertial sensors makes it difficult to determine the system and measurement noise, especially when the unmanned vehicle is maneuvering [23]. In addition, the performance of inertial sensors also leads to unsatisfactory results of the coarse alignment, resulting in the initial misalignment angle not satisfying the small-angle assumption [24]. Therefore, nonlinear and robust state estimation algorithms, such as Extended Kalman filter (EKF), Unscented Kalman filter (UKF), Cubature Kalman filter (CKF), $H_{\infty }$ filter, and Huber filter, have been studied to solve the problems of error model nonlinearity, noise uncertainty, and bad external interference [6,25].

In order to enhance the initial alignment accuracy of low-precision SINS for unmanned vehicles, a novel initial alignment algorithm is proposed in this paper. In this novel initial alignment algorithm, the inertial sensor signal is preprocessed by utilizing an improved EMD denoising method based on Extreme Learning Machine (ELM) [26] and Shannon entropy to eliminate the effect of random noises on the coarse alignment firstly. Furthermore, a robust estimation method is proposed based on CKF and Huber filter to solve the influence of the nonlinearity and uncertainty on the accuracy of the fine alignment. Real-data is used to test the effectiveness of this novel initial alignment algorithm.

The rest is organized as follows. Section 2 indicates the relative background knowledge about initial alignment for unmanned vehicles. In Section 3, a novel initial alignment algorithm based on the improved EMD method and robust Huber filter is presented. The verification results are presented in Section 4 and the conclusions are drawn in Section 5.

## 2. Background Knowledge

### 2.1. Analytical Coarse Alignment Algorithm Based on the Solidification Coordinate Frame

**Figure 1:**
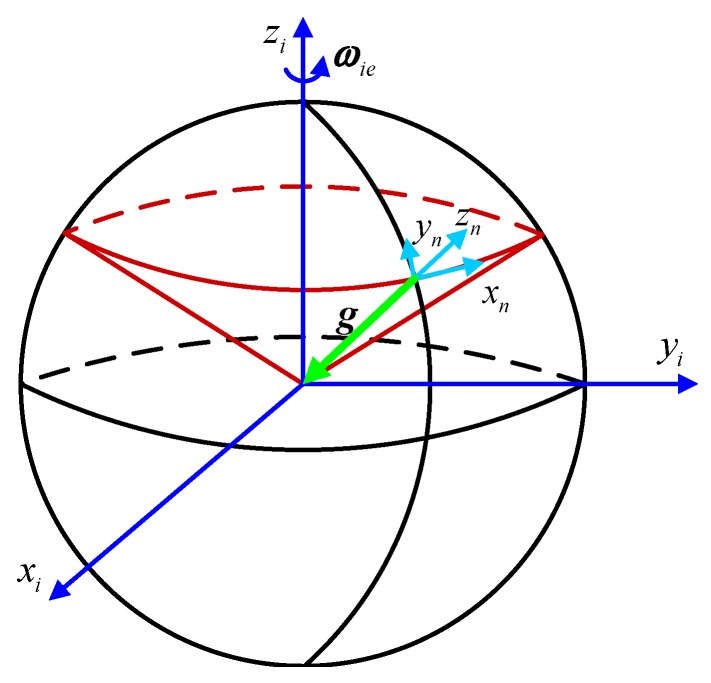
Moving trajectory of gravity vector in the inertial frame.

The initial alignment is a key technology of the unmanned vehicle’s SINS. The speed and accuracy of the initial alignment affect the system’s starting speed and navigation accuracy, respectively. The initial alignment is divided into coarse alignment and fine alignment. Since there is no priori knowledge, only the measurement information from the accelerometer and the gyroscope can be used in the coarse alignment, the most common used coarse alignment method is the analytical method. In the analytical coarse alignment algorithm, a non-collinear vector is constructed firstly by utilizing the gravity vector and the earth’s rotation vector measured by accelerometers and gyroscopes, respectively. Then, the strapdown attitude matrix is calculated analytically. The traditional analytical coarse alignment requires the carrier to be static. However, SINS is inevitably subjected to various disturbance in the alignment process such as gusts, engine vibration, and maneuvering in dynamic conditions when the unmanned vehicle is moving. Thus, to extend the application and improve the accuracy of the coarse alignment, an analytical coarse alignment algorithm based on solidification coordinate frame was proposed. Its basic principle is illustrated as follows: the output of gyroscopes tracks the change of the inertial coordinate frame (*i*-frame), the output of accelerometers is projected on the *i*-frame, and, after isolating the vehicle’s acceleration relative to the Earth, it can be observed that the gravity acceleration *g* due to the earth rotation is slowly drifting in the *i*-frame; the drift of *g* is in a cone in which the earth rotation axis is the main axis, and the geographical north can be determined from the *g* drift. The solidification coordinate frame ($i_{b0}$-frame) is defined as an orthogonal reference frame nonrotating relative to the *i*-frame, which is formed by fixing the body coordinate frame (*b*-frame) at startup in the inertial space. The attitude matrix $C_{b}^{n}$ is time-changing when the unmanned vehicle is moving. The attitude matrix $C_{b}^{n}\left(t\right)$ at time *t* can be decomposed as follows:

(1) $$C_{b}^{n}\left(t\right)=C_{e}^{n}\left(t\right)C_{i}^{e}\left(t\right)C_{i_{b0}}^{i}\left(t\right)C_{b}^{i_{b0}}\left(t\right),$$

where *n*-frame means the navigation coordinate frame and *e*-frame denotes the earth coordinate frame; $C_{e}^{n}$ denotes the coordinate transformation matrix between the *e*-frame and the *n*-frame; $C_{i}^{e}$ is the coordinate transformation matrix between the *i*-frame and the *e*-frame; $C_{i_{b0}}^{i}$ indicates the coordinate transformation matrix between $i_{b0}$-frame and *i*-frame; $C_{e}^{n}$, $C_{i}^{e}$ and $C_{b}^{i_{b0}}$ are known previously and expressed as follows:

(2) $$C_{e}^{n}=\left[\begin{array}{ccc} 0 & 1 & 0\\ -\sin L & 0 & \cos L\\ \cos L & 0 & \sin L \end{array}\right],$$

(3) $$C_{i}^{e}=\left[\begin{array}{ccc} \cos\omega_{ie}\left(t-t_{0}\right) & \sin\omega_{ie}\left(t-t_{0}\right) & 0\\ -\sin\omega_{ie}\left(t-t_{0}\right) & \cos\omega_{ie}\left(t-t_{0}\right) & 0\\ 0 & 0 & 1 \end{array}\right],$$

(4) $${\dot{C}}_{b}^{i_{b0}}=C_{b}^{i_{b0}}\left[\omega_{ib}^{b}\times \right],$$

wherein *L* denotes the local latitude; $\omega_{ie}$ is the Earth’s rotation angular rate; $t_{0}$ denotes the starting time of initial alignment and *t* indicates the present time; $\omega_{ib}^{b}$ is the angular rate that is measured by the gyroscope directly. Since only $C_{i_{b0}}^{i}$ is not determined in Equation (1), the calculation of $C_{b}^{n}$ can be translated into the calculation of $C_{i_{b0}}^{i}$. According to the principle of the double-vector attitude, $C_{i_{b0}}^{i}$ can be determined by two non-collinear vectors whose projections in the *i*-frame and the $i_{b0}$-frame are already known. The moving trajectory of the gravity vector in the *i*-frame is shown as Figure 1. It is known from this figure that the projection in the *i*-frame of the gravity vector trajectory, which, changing with time, is a cone. Since these projections are non-collinear at different time epochs, two non-collinear vectors a and b can be constructed by utilizing the gravity vector and their projections in the *i*-frame are expressed as $a^{i}$ and $b^{i}$. Thus, it is obvious that

(5) $$a^{i_{b0}}=C_{i}^{i_{b0}}a^{i},$$

(6) $$b^{i_{b0}}=C_{i}^{i_{b0}}b^{i}.$$

Define a vector c=a×b satisfying

(7) $$c^{i_{b}}=C_{i}^{i_{b}}c^{i}.$$

$C_{i_{b0}}^{i}$ is calculated from Equations (5)–(7) as

(8) $$C_{i_{b0}}^{i}={\left[\begin{array}{c} {\left(a^{i}\right)}^{⊤}\\ {\left(b^{i}\right)}^{⊤}\\ {\left(c^{i}\right)}^{⊤} \end{array}\right]}^{-1}\cdot \left[\begin{array}{c} {\left(a^{i_{b}}\right)}^{⊤}\\ {\left(b^{i_{b}}\right)}^{⊤}\\ {\left(c^{i_{b}}\right)}^{⊤} \end{array}\right].$$

Therefore, the matrix $C_{b}^{n}$ can be calculated by Equations (1) and (8), accomplishing the coarse alignment based on the solidification coordinate frame. There are two methods to construct the non-collinear vectors in the coarse alignment algorithm based on the solidification coordinate frame, which are velocity-based method and position-based method. In addition, in this manuscript, the position-based method is utilized:

(9) $$\left\{\begin{array}{c} {\tilde{a}}^{i_{b0}}={\tilde{S}}^{i_{b0}}\left(t_{k1}\right)=\int_{t_{0}}^{t_{k1}}{\tilde{V}}^{i_{b0}}\left(t\right)dt,\\ {\tilde{b}}^{i_{b0}}={\tilde{S}}^{i_{b0}}\left(t_{k2}\right)=\int_{t_{0}}^{t_{k2}}{\tilde{V}}^{i_{b0}}\left(t\right)dt. \end{array}\right.$$

${\tilde{C}}_{b}^{i_{b0}}$ is the actual value of $C_{b}^{i_{b0}}$ and obtained from the following equation:

(10) $${\dot{\tilde{C}}}_{b}^{i_{b0}}={\tilde{C}}_{b}^{i_{b0}}\left[{\tilde{\omega }}_{ib}^{b}\times \right],$$

wherein ${\tilde{\omega }}_{ib}^{b}$ is the actual measured value of gyroscopes and expressed as

(11) $${\tilde{\omega }}_{ib}^{b}=\omega_{ib}^{b}+\delta \omega_{ib}^{b},$$

wherein $\delta \omega_{ib}^{b}$ is the measurement uncertainty of gyroscopes. In addition, ${\tilde{V}}^{i_{b0}}\left(t_{kj}\right)$ in Equation (9) is expressed as

(12) $${\tilde{V}}^{i_{b0}}\left(t_{kj}\right)=\int_{t_{0}}^{t_{kj}}{\tilde{C}}_{b}^{i_{b0}}{\tilde{f}}^{b}dt,j=1,2,$$

where ${\tilde{f}}^{b}$ is the actual measured value of accelerometers and expressed as

(13) $${\tilde{f}}^{b}=C_{i_{b0}}^{b}\left[-C_{i}^{i_{b0}}g^{i}\left(t\right)+{\dot{v}}^{i_{b0}}+\omega_{ie}^{i_{b0}}\times v^{i_{b0}}\right]+\delta f^{b},$$

where ${\dot{v}}^{i_{b0}}$ is the unmanned vehicle’s acceleration projected to the $i_{b0}$-frame in the moving base and $v^{i_{b0}}$ is the unmanned vehicle’s velocity projected to the $i_{b0}$-frame; $\delta f^{b}$ is the measurement uncertainty of accelerometers. It is known from the previous analysis that the measurement uncertainty of gyroscopes $\delta \omega_{ib}^{b}$ affects the calculation result of $C_{b}^{i_{b0}}$; and the measurement uncertainty of accelerometers $\delta f^{b}$ affects the calculation result of the gravity vector. Meanwhile, $\delta \omega_{ib}^{b}$ and $\delta f^{b}$ will lead the bias between the calculated value and the expected value of $a^{i_{b0}}$ and $b^{i_{b0}}$, affecting the accuracy of coarse alignment.

### 2.2. Integrated Fine Alignment Algorithm Based on the CKF Method

#### 2.2.1. Nonlinear Model for Integrated Fine Alignment of SINS/ GNSS Integrated Navigation Systems

In order to improve the initial alignment accuracy and speed of the unmanned vehicle’s SINS, the localization information from the external GNSS is provided and the state vector of SINS/GNSS integrated navigation systems is estimated in real time by utilizing estimation methods to complete the fine alignment of the unmanned vehicle. Considering the nonlinear problem in the actual system, the nonlinear model of SINS/GNSS integrated navigation systems is established firstly.

In the literature [27,28], the nonlinear model of SINS/GNSS integrated navigation systems is derived in detail. No longer exhaustive, only the nonlinear equations are given here. The attitude error of SINS is expressed as follows based on the Euler error angle method:

(14) $$\dot{\phi }=C_{\omega }^{-1}\left[\left(I-C_{n}^{n^{\prime }}\right){\hat{\omega }}_{in}^{n}+C_{n}^{n^{\prime }}\delta \omega_{in}^{n}-C_{b}^{n^{\prime }}\varepsilon^{b}\right]+C_{\omega }^{-1}C_{b}^{n^{\prime }}w_{g}^{b},$$

wherein $\phi ={\left[\begin{array}{ccc} \phi_{x} & \phi_{y} & \phi_{z} \end{array}\right]}^{⊤}$ is the Euler error angle vector, $n^{\prime }$ denotes the calculation navigation coordinate system of SINS, and the direction cosine matrix from *n* to $n^{\prime }$ is $C_{n}^{n^{\prime }}$; $C_{b}^{n^{\prime }}$ denotes the direction cosine matrix from *b* to $n^{\prime }$; $\varepsilon^{b}$ and $w_{g}^{b}$ are the gyro constant drift vector and the zero-mean Gaussian white noise vector, respectively; ${\hat{\omega }}_{in}^{n}$ is the gyro measurement vector; $\omega_{in}^{n}$ is the rotating angular rate vector of *n* relative to *i*; $\delta \omega_{in}^{n}$ is the calculated error vector of $\omega_{in}^{n}$. The gyro measurement vector is equal to ${\hat{\omega }}_{in}^{n}=\omega_{in}^{n}+\delta \omega_{in}^{n}$. $C_{\omega }$ is an intermediate matrix as follows:

(15) $$C_{\omega }=\left[\begin{array}{ccc} \cos\phi_{y} & 0 & -\sin\phi_{y}\cos\phi_{x}\\ 0 & 1 & \sin\phi_{x}\\ \sin\phi_{y} & 0 & \cos\phi_{y}\cos\phi_{x} \end{array}\right].$$

The velocity error equation is given by:

(16) $$\begin{array}{ccc} \delta {\dot{v}}^{n} & = & C_{b}^{n^{\prime }}{\hat{f}}^{b}-C_{b}^{n}{\hat{f}}^{b}+C_{b}^{n}\nabla^{b}-\left(2\delta \omega_{ie}^{n}+\delta \omega_{en}^{n}\right)\\ & & \times ({\hat{v}}^{n}-\delta v^{n})-\left(2{\hat{\omega }}_{ie}^{n}+{\hat{\omega }}_{en}^{n}\right)\times \delta v^{n}+C_{b}^{n}W_{a}^{b}, \end{array}$$

wherein ${\hat{f}}^{b}$ denotes the specific force vector; ${\hat{\omega }}_{ie}^{n}$ and ${\hat{\omega }}_{en}^{n}$ are the calculated Earth’s rotating angular rate and calculated angular rate, $\delta \omega_{ie}^{n}$ and $\delta \omega_{en}^{n}$ indicate the error vectors of ${\hat{\omega }}_{ie}^{n}$ and ${\hat{\omega }}_{en}^{n}$, respectively; ${\hat{v}}^{n}$ and $\delta {\hat{v}}^{n}$ denote velocity measurement vector and its corresponding error vector.

The longitude error δλ and the latitude error δφ:

(17) $$\left\{\begin{array}{c} \delta \dot{\lambda }=\delta \varphi \tan\varphi \sec\varphi \frac{v_{x}}{R_{N}}+\sec\varphi \frac{\delta v_{x}}{R_{N}},\\ \delta \dot{\varphi }=\frac{\delta v_{y}}{R_{M}}, \end{array}\right.$$

wherein $R_{M}$ and $R_{N}$ are the Earth’s radii of the meridian circle and the prime vertical circle, respectively; λ and φ are the longitude and latitude of a point of interest; $v_{x}$ and $v_{y}$ are the east and north velocities with their velocity errors $\delta v_{x}$ and $\delta v_{y}$, respectively.

The differential equation of inertial sensors are:

(18) $$\left\{\begin{array}{c} {\dot{\varepsilon }}^{b}=0,\\ {\dot{\nabla }}^{b}=0. \end{array}\right.$$

The state function is expressed as follows:

(19) $$\dot{X}\left(t\right)=f\left[X(t),w(t)\right],$$

where the state vector and the process noise are expressed as follows:

(20) $$\begin{array}{ccc} X(t) & = & {\left[\delta \lambda \;\;\delta \varphi \;\;\delta h\;\;\delta v_{x}\;\;\delta v_{y}\;\;\delta v_{z}\;\;\phi_{x}\;\;\phi_{y}\;\;\phi_{z}\;\;\nabla_{x}\;\;\nabla_{y}\;\;\nabla_{z}\;\;\varepsilon_{x}\;\;\varepsilon_{y}\;\;\varepsilon_{z}\right]}^{⊤}, \end{array}$$

(21) $$\begin{array}{ccc} w(t) & = & {\left[0_{1\times 3}\;\;w_{ax}\;\;w_{ay}\;\;w_{az}\;\;w_{gx}\;\;w_{gy}\;\;w_{gz}\;\;0_{1\times 6}\right]}^{⊤}, \end{array}$$

where Q(t) denotes the covariance matrix of the process noise.

The difference between the localization information provided by GNSS and the localization information calculated by SINS is used as the observation

(22) $$Z\left(t\right)=\left[\begin{array}{c} \delta \lambda \\ \delta \varphi \\ \delta h \end{array}\right]=\left[\begin{array}{c} \lambda^{SINS}-\lambda^{GNSS}\\ \varphi^{SINS}-\varphi^{GNSS}\\ h^{SINS}-h^{GNSS} \end{array}\right]$$

and the observation function is expressed as

(23) Z(t)=H(t)X(t)+η(t),

wherein observation matrix and observation noise are expressed as follows:

(24) $$H\left(t\right)=\left[\begin{array}{cc} I_{3\times 3} & 0_{3\times 12}, \end{array}\right]$$

(25) $$\eta \left(t\right)={\left[\eta_{\lambda }\;\;\eta_{\varphi }\;\;\eta_{h}\right]}^{⊤},$$

where R(t) is the covariance matrix of the observation noise.

#### 2.2.2. Nonlinear Filter Algorithm Based on CKF

As we all know that CKF based on the spherical-radial cubature criterion is one of the most mature practical nonlinear filters. The same as the EKF, it is also based on Bayesian estimation. However, unlike the Taylor expression, which is used for linear approximation in EKF, in CKF, a set of Cubature points and corresponding weights are utilized to approximate the mean and variance of the probability distribution [29]. In this nonlinear filter, the Cubature points and weights are set by $\left[\xi_{i},\omega_{i}\right]$ firstly as follows:

(26) $$\left\{\begin{array}{c} \xi_{i}=\sqrt{N}{\left[1\right]}_{i},\\ \omega_{i}=\frac{1}{2N}, \end{array}\right.$$

wherein $\xi_{i}$ is the *i*-th cubature point and $\omega_{i}$ is the corresponding weight; i=1,2,…,2N, and *N* is the dimension of the nonlinear system.

**Algorithm 1** CKF algorithm
**Require:** k=0, ${\hat{x}}_{0|0}$, ${\hat{P}}_{0|0}$, Q, R**Ensure:** ${\hat{x}}_{k|k}$, ${\hat{P}}_{k|k}$  1:
**if** k≥1 **then**
  2:    Cholesky decomposition of $P_{k-1|k-1}$;  3:    Calculate the Cubature point set $X_{i,k-1|k-1}\;\;\left(i=1,2,\ldots ,2N\right)$;  4:    Propagate the Cubature point by using the state equation $X_{i,k|k-1}^{*}$;  5:    Calculate the prediction of the state ${\hat{x}}_{k|k-1}$;  6:    Calculate the prediction of the state covariance matrix $P_{k|k-1}$;  7:    Cholesky decomposition of $P_{k|k-1}$;  8:    Calculate the Cubature point set again $Y_{i,k|k-1}\left(i=1,2,\ldots ,2N\right)$;  9:    Propagate the Cubature point by using the measurement equation $Y_{i,k|k-1}^{*}$;  10:    Calculate the prediction of the measurement ${\hat{y}}_{k|k-1}$;  11:    Calculate the autocorrelation matrix $P_{k|k-1}^{zz}$;  12:    Calculate the cross-correlation matrix $P_{k|k-1}^{xz}$;  13:    Calculate the filtering gain $K_{k}$;  14:    Calculate the estimation of the state ${\hat{x}}_{k|k-1}$;  15:    Calculate the estimation of the state covariance matrix $P_{k|k}$;  16:
**end if**



Since the CKF is under the Bayesian estimation framework, we suppose that the initial and the prior density are already known accurately. Similar to EKF, the filtering operation can be divided into two parts: the time update and the measurement update [29]. The implementation of the CKF is given in detail as Algorithm 1.

## 3. Initial Alignment Algorithm for the Unmanned Vehicle

### 3.1. Improved Denoising Method Based on the ELM and EMD–Shannon Method

It is known from the analysis results of Section 2.1 that the accuracy of the coarse alignment is affected by the measurement uncertainty of SINS inertial sensors. However, due to a vehicle’s maneuvering, environment disturbance or mechanical noises in actual applications, the output signal is large nonlinearity and contains non-stationary random noises, which seriously affects the measurement accuracy of inertial sensors. In order to improve the accuracy of the coarse alignment, a denoising method based on the ELMEMD-Shannon method is proposed in this paper to preprocess the output signal of inertial sensors.

#### 3.1.1. A Brief Review of the EMD Method

The EMD-based denoising method is one of the most common used signal denoising methods. The EMD-based method relies on the time scale of the signal itself to decompose adaptively multiple times and does not need the basis function. Therefore, the EMD-based method has great advantages in dealing with nonlinear and non-stationary random signals. Considered the output signal characteristics of inertial sensors, the EMD-based method is adopted to denoise the low-precision inertial measurement unit (IMU) signal in this paper.

The EMD, an effective analysis method for nonlinear and non-stationary signals, decomposes the signal into several Intrinsic Mode Functions (IMFs) and a residue adaptively based on the intrinsic characteristics of the signal. The so-called IMF is a function or signal that satisfies the following two conditions:

- In the entire data set, the difference between the number of extreme values and the number of zero crossings must not be greater than one;
- At any point of the data set, the mean value of the envelope defined by the local extrema is all zero.

For a given signal x(t), the process of EMD decomposition is illustrated as follows:

- First of all, all local extreme values of the signal should be found out and identified. The cubic spline line is used to connect all the local maxima and all the local minima, producing the upper envelope and the lower envelope, respectively. Thus, all of the signal data x(t) should be covered by the upper and lower envelopes. We suppose that $m_{1}$ is the mean of the covered data by the envelopes, so the difference between the signal x(t) and the mean $m_{1}$ can be taken as a new signal, indicated as $h_{1}$, named the first component:
  (27) $$h_{1}=x\left(t\right)-m_{1}.$$
- In general, we can not guarantee that $h_{1}$ is a stationary data sequence, so we should repeat the above operation. Now, $h_{1}$ is taken as a new signal $h_{10}$ and its envelope mean is $m_{11}$. Thus, the data sequence after removing the low-frequency components represented by $m_{11}$ is $h_{11}$:
  (28) $$h_{11}=h_{1}-m_{11}.$$
  Repeating the above operation up to *k* times, we can obtain the signal and the first IMF as follows:
  (29) $$\left\{\begin{array}{c} h_{1k}=h_{1(k-1)}-m_{1k},\\ imf_{1}=h_{1k}. \end{array}\right.$$
- Finally, the residual is a new signal that removed the high frequency component from the original signal:
  (30) $$r_{1}=x\left(t\right)-imf_{1}.$$
- Then, we can deal with the residual iteratively to get the other IMFs. The stop iterating condition is that when the residue $r_{n}$ becomes a monotonic function or a function with only one extremum. It means that no more IMF can be extracted from the residual signal $r_{n}$. Thus, after the decomposition, x(t) is decomposed into several IMFs and a residual:
  (31) $$x\left(t\right)=\sum_{i=1}^{n}imf_{i}+r_{n}.$$

#### 3.1.2. Improved EMD Denoising Method Based on ELM and Shannon Entropy

Due to the uncertainty of the basis function, the EMD method has the endpoint effect when decomposing the limited signal sequence, which will seriously affect the reliability of the EMD method. Instead of training the parameters, the ELM method uses the minimum-norm least-squares solution as the output weight of the network by solving the linear equations [30]. Thus, the ELM method has faster speed and better performance. Therefore, in order to solve the endpoint effect of the traditional EMD denoising method, the ELM method is utilized to predict and extend the inertial sensor data in this paper.

According to the basic properties of the EMD decomposition, the active ingredient concentration of signal increases with the index of IMF. The first few IMFs, especially the first IMF component, consist almost entirely of high-frequency noise. As a result, there should be a sudden change in the ratio of the high-frequency noise and the effective signal in the IMF components, resulting in a sudden change in the probability distribution of the IMF component. The effective signal can be separated by determining this mutation point.

The Shannon entropy is a method to quantify the information. Suppose that the probability distribution of a discrete variable is $\left(p_{1},p_{2},\ldots ,p_{n}\right)$ and its Shannon entropy is defined as

(32) $$S=-\sum_{i=1}^{n}p_{i}\log\left(p_{i}\right).$$

Suppose that x(t) is the sampled signal and it is expressed as

(33) x(t)=y(t)+z(t),

where y(t) is the original signal and z(t) is the noise signal.

The original signal has been decomposed into *n* IMFs and a residual by utilizing the EMD method. In order to reduce the mixing of noise, only a few IMFs and residuals are superimposed in the process of signal restoring by using the IMFs:

(34) $$x\left(t\right)=\sum_{j=k}^{n}imf_{j}\left(t\right)+r_{n}\left(t\right),k=2,\ldots ,n.$$

A noise separation method based on the Shannon entropy is proposed in this paper. Suppose that the entropy of each IMF is $S_{i}\left(i=1,2,\ldots ,n\right)$ and the entropy variation between adjacent IMFs is expressed as:

(35) $$\Delta S_{i}=S_{i+1}-S_{i}\left(i=1,2,\ldots ,n-1\right).$$

Thus, the corresponding index $j_{s}$ can be expressed as

(36) $$j_{s}=\underset{1\leq i\leq n-1}{\arg\max}\left(\Delta |S_{i}|\right).$$

Therefore, an improved EMD denoising method based on the ELM method and the Shannon entropy is proposed in this paper. In this novel algorithm, the IMU signal is decomposed by using the ELMEMD method to obtain a series of IMFs firstly; then, after calculating the Shannon entropy of each IMF, the index corresponding to the maximum adjacent Shannon entropy variation is determined; on this basis, the inertial sensor signal is reconstructed so as to effectively suppress the influence on the signal quality of the inertial sensor.

**Figure 2:**
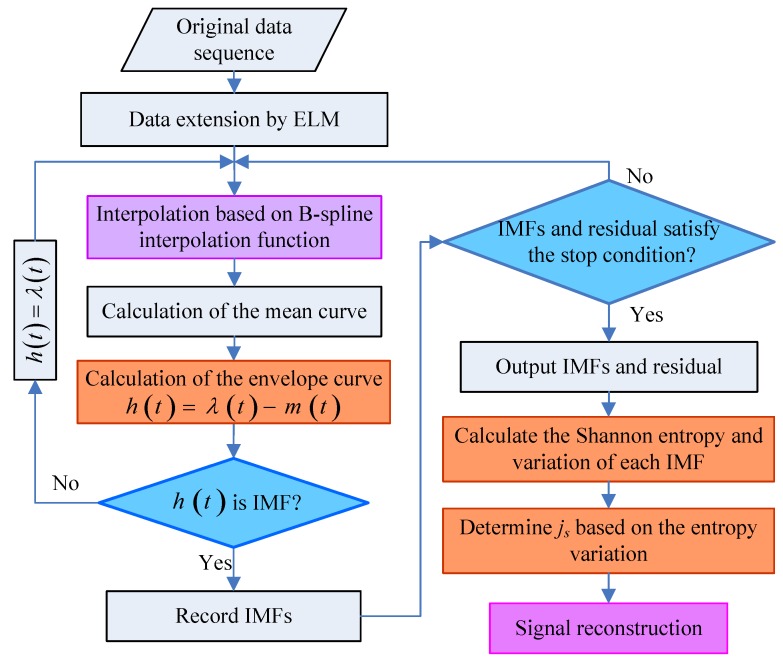
The process of IMU signal denoising based on ELMEMD.

The process of this novel denoising method is shown as Figure 2.

The specific steps of the denoising method are as follows:

Step 1: Extend the time series to the right and seven adjacent samples are used as the input of the ELM method. Use the adjacent right (or left) samples as a training sample.
Step 2: Add the previous prediction value into each new learning before each step of learning. Repeatedly training and learning, obtain all the required extension sequence according to the required extension of the extreme points.
Step 3: Decompose the inertial sensor signal into several IMFs and residuals by using the EMD method.
Step 4: Calculate the Shannon entropy of each IMF $S_{i}$.
Step 5: Calculate the adjacent Shannon entropy variation $\Delta S_{i}$.
Step 6: Determine the value of $j_{s}$ based on Step 5.
Step 7: Reconstruct the signal based on the value of $j_{s}$.

### 3.2. Improved Robust Filter based on the RHCKF Method for Fine Alignment

The filtering performance directly affects the estimation accuracy of the system state vector. Compared with the commonly used filters, such as KF, EKF, and UKF, CKF can not only be used in nonlinear systems but also obtain better filtering accuracy. However, as we all know, the CKF is based on Bayesian estimation. When the system model is known exactly in advance and the external noise signal is a Gaussian noise, the CKF can obtain the optimal estimations; otherwise, the State estimation may be suboptimal. Because of vehicle’s maneuvering and external airflow impact, the condition of optimal estimation is difficult to ensure in practical applications, resulting in filtering accuracy decreasing or even filtering divergence. From the perspective of the approximate Bayesian estimation, the essence of the Huber filter is adding a weight matrix before the innovation, to truncate the average of filter innovations, thereby suppressing the effect of interference noise or outliers in system observation information and enhancing its robustness [22,31,32]. Assumed in a nonlinear system, the transformation innovation probability density based on the Huber cost function can be used to calculate the transformation innovation $\eta_{k}$

(37) ηk=Pzz,kk-1-1122zk-z^kk-1,

wherein $P_{zz}$ is the autocorrelation covariance matrix of the observation, $z_{k}$ and ${\hat{z}}_{k|k-1}$ are the actual observation and the predicted observation. Calculating the transformation innovation function $\varphi (\eta_{k,i})$

(38) φηk,i=ζi,ηk,i<γ,sgnηk,iγζiζiηk,iηk,i-0.5γ2,ηk,i>γ,

wherein γ is the adjustment factor of the cost function in the Huber filter, and the intermediate variable $\zeta_{i}$ can be calculated by the following equation:

(39) ζi2=2πerfγγ22+4exp-γ2-γ222γ-1+γ-32πerfγγ22+2exp-γ2-γ222γ-1.

In Equation (39), erf(x) is the error function and:

(40) $$erf\left(x\right)=\frac{2}{\sqrt{\pi }}\int_{0}^{x}e^{-t^{2}}dt.$$

Then, the innovation weight matrix $\Theta_{k}$ and ρ(k) can be obtained:

(41) $$\left\{\begin{array}{c} \Theta_{k}=diag\left[\varphi \left(\eta_{k,i}\right)\right],\\ \rho \left(k\right)=\left(1-\varepsilon \right)\left(1-2\Phi \left(-k\right)\right), \end{array}\right.$$

wherein 0≤ε≤1 and is a standard Gaussian distribution function. The state vector and the state covariance matrix can be estimated by Equation (42), suppressing the effect of outliers and accomplishing the high-precision robust estimation:

(42) $$\left\{\begin{array}{c} {\hat{x}}_{k\left|k\right.}={\hat{x}}_{k\left|k-1\right.}+P_{k\left|k-1\right.}P_{zz,k\left|k-1\right.}^{-1}\Theta_{k}\left(z_{k}-{\hat{z}}_{k\left|k-1\right.}\right),\\ P_{k\left|k\right.}=P_{k\left|k-1\right.}-P_{xz,k\left|k-1\right.}P_{zz,k\left|k-1\right.}^{-1}P_{xz,k\left|k-1\right.}^{T}\rho \left(k\right). \end{array}\right.$$

**Figure 3:**
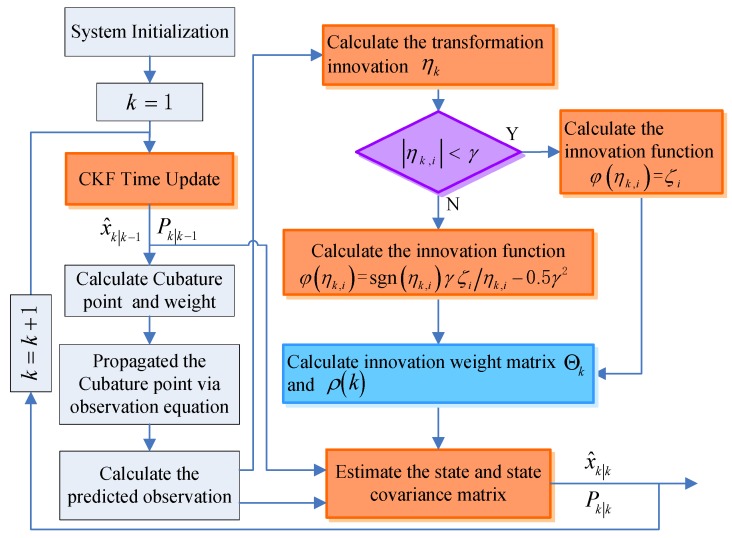
The flow chart of the RHCKF method.

Therefore, the Huber filter is introduced into the CKF method and an improved robust Huber-CKF (RHCKF for short) algorithm is constructed in this manuscript. The flow chart of this improved algorithm is shown in Figure 3. In this improved algorithm, the Huber filter is used to preprocess observations based on CKF framework, and a CKF measurement update is used to complete the nonlinear system estimation, avoided the linearized approximation of nonlinear equations and implemented the robustness of the algorithm.

### 3.3. Improved Initial Alignment Algorithm Based on ELMEMD-Shannon and RHCKF Methods

The initial alignment is a prerequisite for the normal operation of the unmanned vehicle’s SINS and the alignment accuracy and convergence speed are two important performance indicators. Due to the limitation of installation space and weight in unmanned vehicles, the inertial sensor is generally smaller and low accurate, which are easily interfered by external factors, especially under dynamic conditions. In order to improve the alignment performance of unmanned vehicles in dynamic base, this paper presents a novel initial alignment algorithm. Firstly, the signal of inertial sensors is denoised by the ELMEMD-Shannon method, and, on this basis, a coarse alignment based on the solidification coordinate frame is used, suppressing the alignment error caused by dynamic noises; secondly, nonlinear system equations of the SINS/GNSS integrated fine alignment are established; meanwhile, the RHCKF filter is used to complete the state estimation, inhibited the impact of system nonlinearity and uncertainty, and ultimately improved the alignment accuracy and robustness of unmanned vehicles under dynamic conditions. The relative pseudo-code is illustrated as Algorithm 2.

**Algorithm 2** Improved Initial Alignment Algorithm
**Require:** k=0; Coarse alignment time $T_{coarse}$; Total alignment time *T*  1:
**if** k≥1 **then**
  2:    **if** $k<T_{coarse}$ **then**  3:        Denoise the inertial sensors’ information with the ELMEMD-Shannon method;  4:        Coarse alignment based on solidification coordinate frame;  5:    **else** $T_{coarse}<k<T$  6:        Fine alignment with the RHCKF filter;  7:    **end if**  8:
**end if**
  9:**return** Output the alignment results


## 4. Test Result and Analysis

### 4.1. Static Test in the Laboratory

#### 4.1.1. Test Environment Establishment

**Figure 4:**
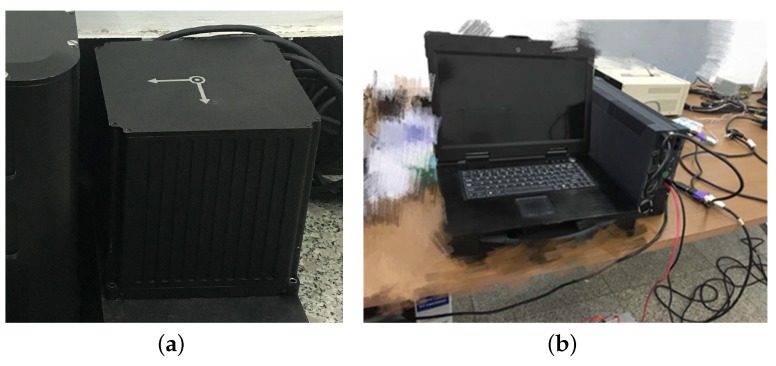
Static test in the laboratory. In this test, a FOG IMU, developed by the Navigation Instrument Institute, Harbin Institute of Technology, is used as shown in (**a**). This FOG IMU is placed on a stable and leveled marble platform to isolate the influence from external disturbance; In (**b**), a rugged laptop is used to collected the source date.

In order to verify the performance of the denoising method proposed in this paper, the static test in the lab has been done firstly. A fiber optic gyro (FOG) IMU, developed by the Navigation Instrument Institute, Harbin Institute of Technology, was used in this test, as shown in Figure 4a. In order to isolate the interference from external environment, the IMU was installed on a metal plate and placed on a vibration isolation platform. The source data was collected by a laptop, shown as Figure 4b. The sampling frequency was 100 Hz and the test lasted 90 min. The source data was processed offline to verify the effectiveness of the proposed algorithm.

#### 4.1.2. Static Test Results and Analysis

**Figure 5:**
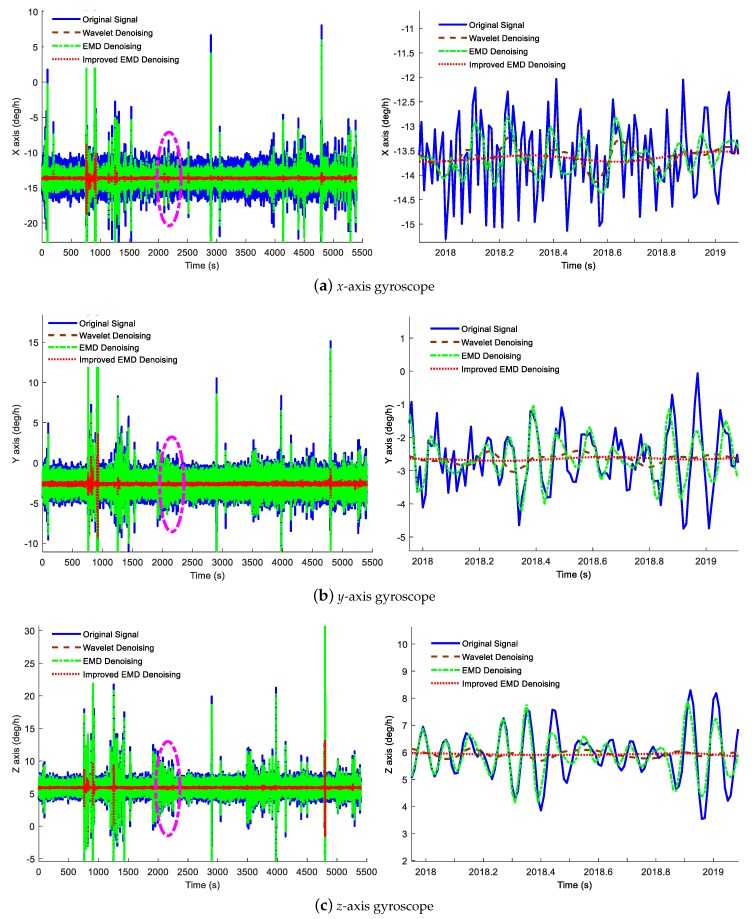
Gyroscope denoising result by utilizing multiple denoising methods. The left subfigures are the denoising results of the *x*-axis, *y*-axis and *z*-axis gyroscopes and the right subfigures are the local magnifications of the purple elliptical regions.

**Figure 6:**
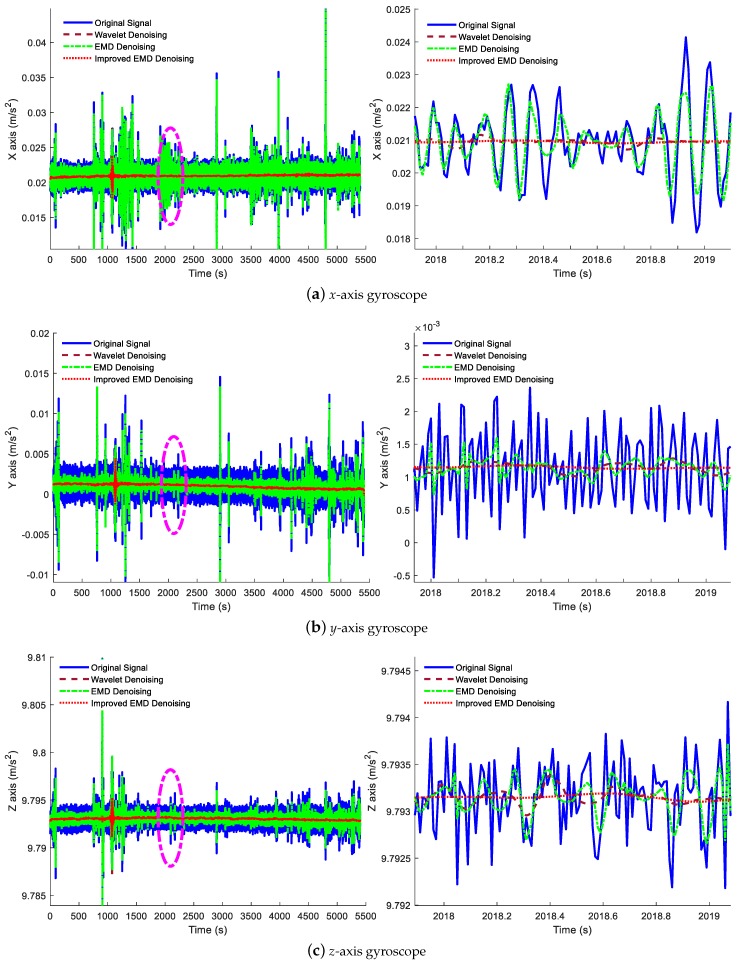
Accelerometers’ denoising result by utilizing multiple denoising methods. The left subfigures are the denoising results of the *x*-axis, *y*-axis and *z*-axis accelerometers and the right subfigures are the local magnifications of the purple elliptical regions.

In this part, three different denoising methods, including the wavelet denoising method, traditional EMD denoising method and the improved ELMEMD-Shannon denoising method, are used to process the static data, verifying the performance of the proposed denoising method. In addition, the denoising results are shown in Figure 5 and Figure 6. In these figures, the blue solid line indicates the original signal, the red-brown dashed line represents the signal with the wavelet denoising method, and the green dot-dashed line represents the signal with the traditional EMD denoising method while the red dotted line denotes the signal with the proposed ELMEMD-Shannon (improved EMD for short) denoising method. In order to analyze the noise reduction effect of the signal more intuitively, the result was locally amplified from 2018 s to 2019 s shown in the corresponding right subfigures of Figure 5 and Figure 6.

It is obviously known from Figure 5 and Figure 6 that amplitudes of inertial sensor signals are decreased with these three denoising methods while the ones of three axes are all much smaller with the improved ELMEMD-Shannon denoising method than the ones with the other two methods.

In order to quantitatively analyze the performance of the proposed denoising method, the Allan variance of gyro signals is calculated and corresponding errors, including the quantizing noise (QN), Random angle walk (RAW), Bias instability (BI), Angular rate walk (ARW) and Rate Ramp (RR), are shown in Table 1, Table 2 and Table 3.

From Table 1, Table 2 and Table 3, errors of original signals are the largest while ones with these three denoising methods are reduced to some extent and errors with ELMEMD-Shannon denoising method is the smallest. Thus, it is clear that gyroscopes’ noise can be eliminated with these three denoising methods, and, moreover, the ELMEMD-Shannon denoising method has better performance.

### 4.2. Dynamic Test in Vehicle

#### 4.2.1. Test Environment Establishment

**Figure 7:**
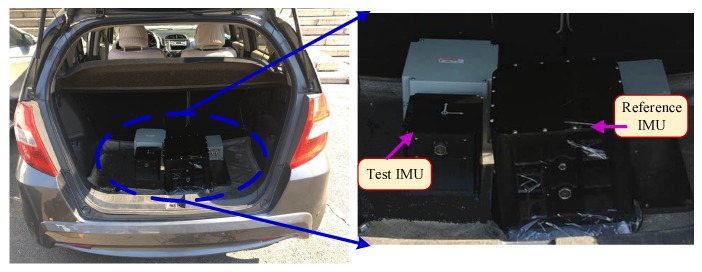
Dynamic test in a car. In this test, a high-precision FOG SINS was used as a reference to provide the attitude reference while a low-accuracy FOG IMU was used to be tested. The sampling frequency is 100 Hz and the testing time is about 1 h.

In order to test the performance of the novel initial alignment algorithm, a dynamic test platform with a test vehicle has been established to simulate the unmanned vehicle dynamic environment, shown as Figure 7. Besides the Global Positioning System (GPS), two FOG IMUs developed by our own lab were used in this test. As shown in Figure 7, a high-precision FOG SINS was used as a reference to provide the attitude reference while a low-accuracy FOG IMU was used to be tested. In this experiment, the initial latitude is 45.7347${}^{\circ }$ N, the total time and the sampling frequency are 1 h and 100 Hz, respectively.

#### 4.2.2. Result and Analysis

**Figure 8:**
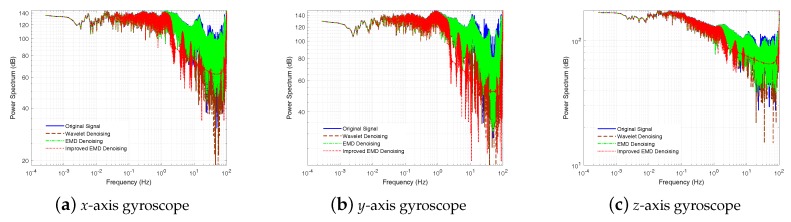
Power spectrum of gyroscope by uitilizing muliple denoising methods.

**Figure 9:**
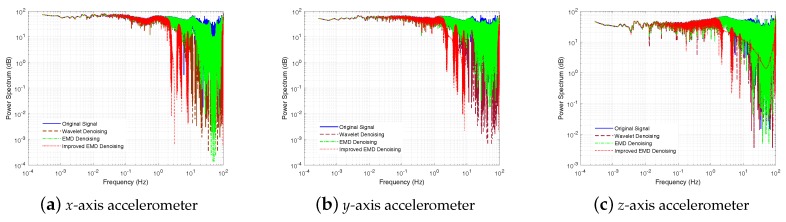
Power spectrum of accelerometers by utilizing multiple denoising methods.

In this part, three denoising methods, the Wavelet denoising method, the EMD denoising method, and the ELMEMD-Shannon denoising method, were used to preprocess original signals firstly. In addition, the power spectrum of gyroscope and accelerometer signals is given, shown in Figure 8 and Figure 9. From them, we can see that the power spectrum at high frequency with the ELMEMD-Shannon denoising method is smaller than the ones with other methods. This means that the high frequency noise can be eliminated with the proposed denoising method in this experiment.

In order to verify the robustness of the proposed initial alignment algorithm, different measurement noise is added to the system measurements, simulating the measurement uncertainty as follows:

(43) $$R\left(t\right)=\left\{\begin{array}{c} 10R_{0};\;\;\;\;\;850\;s<t<1600\;s,\\ 0.4R_{0};\;\;\;\;2000\;s<t<2600\;s, \end{array}\right.$$

where $R_{0}$ is the initial measurement noise matrix.

To verify the performance of the proposed initial alignment algorithm, the initial alignment results were analyzed. Firstly, the analytical coarse alignment method based on the solidification coordinate frame is carried out by utilizing the denoised inertial information; and then two fine alignment algorithms are compared. One algorithm is the fine alignment based on the CKF method while the other is the fine alignment based on the RHCKF method, called ‘Algorithm 1’ and ‘Algorithm 2’, respectively. The time of the coarse alignment was set as two minutes and the fine alignment is carried out for the rest of the time.

**Figure 10:**
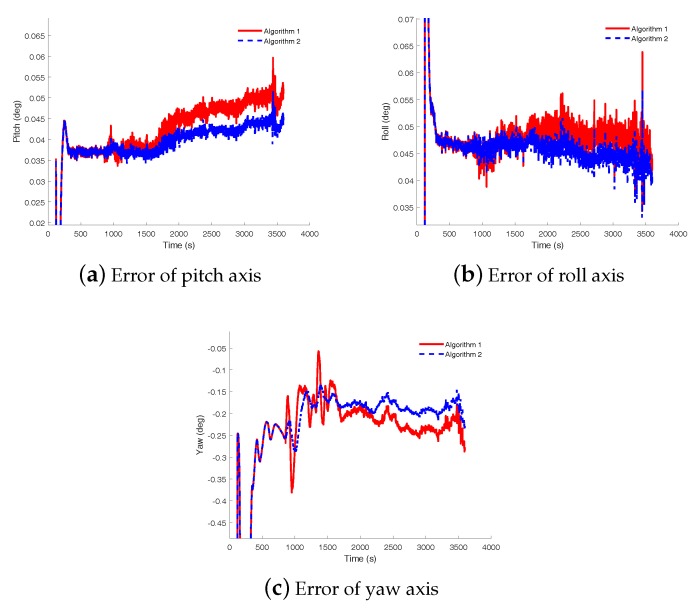
Attitude error curves with different initial alignment algorithms. (**a**,**b**) are the error comparisons of the horizontal attitude angles and (**c**) is the error comparison of the azimuth angles. The red solid line denotes the angle error curve with the CKF fine alignment algorithm while the blue dashed line denotes the angle error curve with the RHCKF fine alignment algorithm.

**Figure 11:**
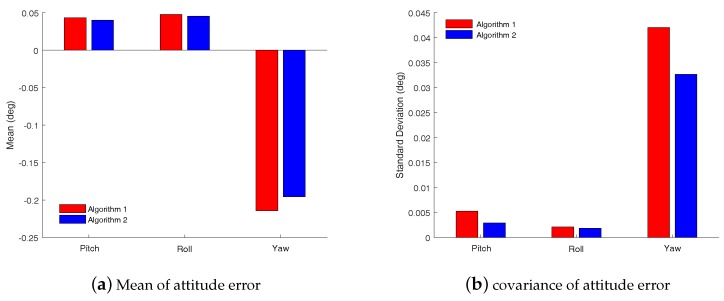
Attitude error statistical property with different initial alignment algorithms. (**a**) is the mean of the pitch, roll and yaw angle errors and (**b**) is the standard deviation of the the pitch, roll and yaw angle errors. The red bar denote the errors obtained by utilizing Algorithm 1 while the blue bar denotes the errors obtained by utilizing Algorithm 2.

The initial alignment errors are shown in Figure 10, and the error curves of the pitch, roll and yaw angle are shown in Figure 10a–c, respectively. In these subfigures, the red solid line denotes the angle error curve with the CKF fine alignment algorithm while the blue dashed line denotes the angle error curve with the RHCKF fine alignment algorithm. From Figure 10, it is obvious that the errors with Algorithm 1 is larger than the ones with Algorithm 2, and, especially, the yaw error with Algorithm 1 is more volatile. In order to further analyze the accuracy of the alignment algorithm, means and standard deviations of the errors from 1000 s to the end in each axis were calculated as shown in Figure 11. Considered the mean and standard deviation of the horizontal attitude angles, although Algorithm 1 and Algorithm 2 have little difference, the errors are smaller with Algorithm 2. Focused on the azimuth angle, the mean and standard deviation are $-0.215^{\circ }$ and $0.042^{\circ }$ with Algorithm 1 while the mean and standard deviation are $-0.196^{\circ }$ and $0.033^{\circ }$, respectively. Thus, we can see that the initial alignment algorithm based on the robust RHCKF method can achieve better performance.

## 5. Conclusions

In order to enhance the accuracy of SINS initial alignment for the unmanned vehicle, a novel initial alignment algorithm is proposed in this manuscript. Considering the influence of high frequency noise from inertial sensors on the coarse alignment accuracy, a signal denoising algorithm based on the ELMEMD method and Shannon entropy for inertial sensors is proposed firstly to reduce the impact of high frequency noises. Considering the influence of the system model uncertainty caused by the maneuvering on the fine alignment accuracy, a novel fine alignment based on the robust RHCKF method to enhance the accuracy and robustness of the fine alignment. The test results showed that the proposed initial alignment algorithm is significantly superior to original algorithms.

## Figures and Tables

**Table 1:** Errors of the *x*-axis gyro signal.

	Orignal	Wavelet	EMD	ELMEMD-Shannon
QN (deg/h)	0.00827	0.00202	0.00599	0.00012
RAW (deg/$h^{1/2}$)	0.00277	0.00056	0.00182	0.00010
BI (deg/h)	0.28702	0.13535	0.09335	0.07795
ARW (deg/h/$h^{1/2}$)	0.58961	1.24727	0.40130	0.33749
RR (deg/h/h)	1.53834	0.72803	0.49311	0.41639

**Table 2:** Errors of the *y*-axis gyro signal.

	Orignal	Wavelet	EMD	ELMEMD-Shannon
QN (deg/h)	0.00785	0.00209	0.00663	0.00020
RAW (deg/$h^{1/2}$)	0.00245	0.00058	0.00198	0.00012
BI (deg/h)	0.20602	0.13690	0.09408	0.09006
ARW (deg/h/$h^{1/2}$)	0.59640	0.89345	0.40294	0.39058
RR (deg/h/h)	1.10144	0.73614	0.49505	0.48154

**Table 3:** Errors of the *z*-axis gyro signal.

	Orignal	Wavelet	EMD	ELMEMD-Shannon
QN (deg/h)	0.00743	0.00170	0.00617	0.00037
RAW (deg/$h^{1/2}$)	0.00226	0.00046	0.00185	0.00016
BI (deg/h)	0.19115	0.13484	0.09195	0.08868
ARW (deg/h/$h^{1/2}$)	0.58718	0.82919	0.39492	0.42828
RR (deg/h/h)	1.02223	0.72478	0.48544	0.52824
